# Identification of targets of JS-K against HBV-positive human hepatocellular carcinoma HepG2.2.15 cells with iTRAQ proteomics

**DOI:** 10.1038/s41598-021-90001-3

**Published:** 2021-05-17

**Authors:** Zhengyun Liu, Yan Xu, Wanling Zhang, Xinghong Gao, Guo Luo, Hong Song, Jie Liu, Huan Wang

**Affiliations:** 1 Key Laboratory of Infectious Disease and Biosafety, Provincial Department of Education, Guizhou, Zunyi Medical University, Zunyi, 563000 Guizhou China; 2 Department of Microbiology, Zunyi Medical University, Zunyi, 563000 Guizhou China; 3 Key Lab for Basic Pharmacology of Ministry of Education and Joint International Research Laboratory of Ethnomedicine, Zunyi Medical University, Zunyi, 563000 Guizhou China

**Keywords:** Cancer, Cell biology, Drug discovery, Microbiology, Molecular biology

## Abstract

JS-K, a nitric oxide-releasing diazeniumdiolates, is effective against various tumors. We have discovered that JS-K was effective against Hepatitis B virus (HBV)-positive HepG2.2.15 cells. This study used iTRAQ to identify differentially expressed proteins following JS-K treatment of HepG2.2.15 cells. Silenced Transgelin (shTAGLN-2.15) cells were constructed, and the cell viability was analyzed by the CCK8 assay after treatment with JS-K. There were 182 differentially expressed proteins in JS-K treated-HepG2.2.15 cells; 73 proteins were up-regulated and 109 proteins were down-regulated. These proteins were categorized according to GO classification. KEGG enrichment analysis showed that Endocytosis, Phagosome and Proteoglycans were the most significant pathways. RT-PCR confirmed that the expression levels of TAGLN, IGFBP1, SMTN, SERPINE1, ANXA3, TMSB10, LGALS1 and KRT19 were significantly up-regulated, and the expression levels of C5, RBP4, CHKA, SIRT5 and TRIM14 were significantly down-regulated in JS-K treated-HepG2.2.15 cells. Western blotting confirmed the increased levels of USP13 and TAGLN proteins in JS-K treated-HepG2.2.15 cells. Molecular docking revealed the binding of JS-K to TAGLN and shTAGLN-2.15 cells were resistant to JS-K cytotoxicity, suggesting that TAGLN could be an important target in JS-K anti-HBV-positive liver cancer cells. These proteomic findings could shed new insights into mechanisms underlying the effect of JS-K against HBV-related HCC.

## Introduction

Hepatitis B virus (HBV) infection is associated with an increased risk of hepatic cirrhosis, hepatic decompensation, and hepatocellular carcinoma (HCC). HCC is one of the leading malignant deaths worldwide. Over 350 million people are chronically infected with HBV who are at greater risk of HCC compared with uninfected people^1^. Therefore, it is very important to find effective drugs to kill HBV-positive HCC.

JS-K (O2-(2, 4-dinitrophenyl) 1-[(4-ethoxycarbonyl) piperazin-1-yl] diazen -1-ium-1, 2-diolate) belongs to the diazeniumdiolate class of prodrug designed to be activated for nitric oxide (NO) release by glutathione S-transferase (GST)^2^. As a novel anticancer drug, JS-K is effective against a variety of cancer cells including human myeloid leukemia cells^3^, human multiple myeloma cell lines^4^, renal cancer cells, prostate cancer cells, brain cancer cells, etc.^5^. In addition, studies from ours and other research groups found that JS-K was also effective against human HCC^6,7^. Our previous study demonstrated that JS-K decreased the expression of HBsAg and HBeAg in HepG2.2.15 cells^8^ and was effective against HBV-positive HCC HepG2.2.15 cells and its mechanism is related with DNA damage and apoptosis^9^.

To better understand the mechanisms of JS-K on HepG2.2.15 cells, we utilized recent advances in proteomics technology and bioinformatics to explore differentially expressed proteins (DEPs), and protein profile associated with HBV-modulated DNA methylation^10^. In the current study, we used stable iTRAQ to identify DEPs in HepG2.2.15 cells treated with or without JS-K. The research will provide novel insights into the molecular mechanisms involved in the anti-HBV and anti-HBV-positive HCC activity of JS-K.

## Materials and methods

### Reagents

JS-K was purchased from Santa Cruz Biotechnology (California, USA); Dulbecco’s Modified Eagle’s Medium/Nutrient Mixture F-12 Ham (DMEM/F12) was obtained from Hyclone (Utah, USA); Fetal bovine serum (FBS) was purchased from Gibco (California, USA); USP13 and TAGLN antibodies were purchased from Santa Cruz Biotechnology (California, USA); β-actin antibody was purchased from BIOSS (Beijing, China); ECL reagents were purchased from Solarbi (Beijing, China); PrimeScript RT reagent Kit and TB Green Premix Ex Taq were purchased from Takara (Tokyo, Japan). CCK8 reagent was purchased from Living (Beijing, China).

### Cell culture and treatment

The HBV-positive human hepatoma cell line HepG2.2.15 cells, shNC-2.15 cells and shTAGLN1^#^-2.15 cells (preserved in Key Laboratory of Infectious Disease & Biosafety, Provincial Department of Education, Guizhou, Zunyi Medical University) were maintained with DMEM/F12 medium containing 10% FBS, 0.5 mg/ml G418, 100 μg/mLstreptomycin and 100 IU/mL penicillin at 5% CO_2_, 37 °C. The medium was changed two times per week. For iTRAQ-based quantitative proteomics analysis, HepG2.2.15 cells were treated 24 h with 10 µM JS-K. The experiments were repeated with at least three different biological replicates. Cell pellets were washed with ice-cold PBS, instantly frozen and stored at − 80 °C until further analysis.

### Protein preparation

For each sample, 6300 μg of total proteins were diluted in 4% SDS, 100 mM Tris–HCl (pH 8.0), and 100 mM DTT and heated at 100ºC for 5 min. Each sample was then cooled to RT(room temperature) and loaded onto an ultrafiltration filter (30 kDa cutoff, Sartorius, Germany) containing 200 μL of UA buffer (8 M urea, 150 mM Tris–HCl, pH 8.0) followed by centrifugation at 14,000×*g* for 30 min and an additional washing step with 200 μL of UA buffer. 100 µL of 50 mM iodoacetamide in UA buffer was subsequently added to the filter to block the reduced cysteine residues, and the samples were then incubated for 30 min at RT in the dark followed by centrifugation at 14,000×*g* for 30 min. The filters were washed with 100 μL of UA buffer and centrifuged at 14,000×*g* for 20 min, and 100 µL dissolution buffer was added to elute protein. The procedure was repeated twice. The protein suspensions were then digested with 40 μL of trypsin (Promega, Madison, WI, USA) buffer (2 μg trypsin in 40μL dissolution buffer) at 37 °C for 16–18 h. Finally, the filter unit was transferred to a new tube and centrifuged at 14,000×*g* for 30 min. The resulting peptides were collected as a filtrate, and the peptide concentration was determined at absorbance of 280 nm^11^.

### iTRAQ labeling and high-pH reversed-phase fractionation

iTRAQ labeling was performed according to the manufacturer's instructions (Applied Biosystems). Briefly, the peptide mixtures were reconstituted with 30 μL of iTRAQ dissolution buffer. The label method of every sample (100 μg) used iTRAQ Reagent-8plex Multiplex Kit (AB SCIEX) (JS-K 0-1, 113; JS-K 0-2, 114; JS-K 0-3, 115; JS-K 10-1, 116; JS-K 10-2, 117; JS-K 10-3, 118). The labeling solution reaction was then incubated at room temperature for 1 h prior to further analysis. The six labeled samples were pooled into one sample and dried in a vacuum centrifuge at room temperature. The iTRAQ-labeled peptides were subjected to High-pH Reversed-Phase Fractionation in 1100 Series HPLC Value System (Agilent) equipped with a Gemini-NX (Phenomenex, 00F-4453-E0) column (4.6 × 150 mm, 3 µm, 110 Å)^12^. The peptides were eluted at a flow rate of 1 mL/min with Buffer A (10 mM ammonium acetate, pH 10.0). The following gradients were applied to perform separation: 100% Buffer A for 30 min, 0–5% Buffer B (10 mM ammonium acetate, 90% v/v ACN, pH 3.0) for 5 min, 5–35% Buffer B for 30 min, 35–70% Buffer B for 10 min, 70–100% Buffer B for 5 min, 100% Buffer B for 15 min, and finally 100% Buffer A for 15 min. The elution process was monitored by measuring absorbance at 214 nm, and fractions were collected every 1 min. The collected fractions (approximately 50) were finally combined into fifteen pools. Each fraction was concentrated via vacuum centrifugation and reconstituted in 40 μL of 0.1% v/v trifluoroacetic acid. All samples were stored at – 80 °C until LC–MS/MS analysis^13^.

### LC–MS/MS analysis

The iTRAQ-labeled samples were analyzed using Easy-nLC nanoflow HPLC system connected to Orbitrap Elite mass spectrometer (Thermo Fisher Scientific, San Jose, CA, USA). A total of 1 μg of each sample was loaded onto Thermo Scientific EASY column using an autosampler at a flow rate of 150 nL/min. The sequential separation of peptides on Thermo Scientific EASY trap column and analytical column was accomplished using a segmented 1 h gradient from Solvent A (0.1% formic acid in water) to 35% Solvent B (0.1% formic acid in 100% ACN) for 50 min, followed by 35–90% Solvent B for 3 min and then 90% Solvent B for 7 min. The column was re-equilibrated to its initial highly aqueous solvent composition before each analysis. The mass spectrometer was operated in positive ion mode, and MS spectra were acquired over a range of 300–2000 m/z. The resolving powers of the MS scan and MS/MS scan at 200 m/z for the Orbitrap Elite were set as 60,000 and 15,000, respectively^13^.

### Protein identification and quantification

The raw files were analyzed using the Mascot 2.2 and Proteome Discoverer 2.1 software (Thermo Fisher Scientific). The Uniprot database was used for this time: P16033_Human_160524_20160514, protein sequence number 160524. The MASCOT search parameters: type of search: MS/MS ion search; enzyme: trypsin; mass values: monoisotopic; max missed cleavages: 2; fixed modifications: carbamidomethyl(c), iTRAQ8plex (N-term), iTRAQ8plex(K); variable modifications: oxidation(M); peptide mass tolerance: ± 20 ppm; instrument type: ESI-TRAP; fragment mass tolerance: 0.1 Da; protein mass: unrestricted; decoy database pattern: true.

### Bioinformatics analysis

Proteins were categorized according to their biological process, molecular function and cellular localization, and functional analysis of proteins identified was conducted using Gene Ontology (GO) annotation (http://www.geneontology.org/). The differentially accumulated proteins were further assigned to the Clusters of Orthologous Groups of proteins (COG) database (http://www.ncbi.nlm.nih.gov/COG/) and the Kyoto Encyclopedia of Genes and Genomes (KEGG) database (http://www.genome.jp/kegg/pathway.html)^14^. GO and pathway enrichment analysis were performed to determine the functional subcategories and metabolic pathways that the differentially accumulated proteins were significantly enriched. TreeView version 1.6 (https://treeview.software.informer.com/1.6/) was used to generate a heatmap^15^.

### RT-PCR analysis

After treatment with JS-K 10 µM for 24 h, total RNA was extracted using Trizol reagent, the cDNA was produced by reverse-transcription of the total RNA employing the PrimeScript RT reagent Kit (TaKaRa, Dalian, China). A Bio-Rad CFX96 System (Bio-Rad, California, USA) was used for quantitative RT-PCR using gene-specific primers. The primer sequences of the tested genes are shown in Supplementary Table S1. The 2^−ΔΔct^ method was used to analyze the relative changes in gene expression. The experiments were repeated three times.

### Western blot analysis

After treatment with JS-K 10 µM 24 h, protein was extracted using protein lysis buffer. The protein samples were resolved using 10% SDS–polyacrylamide gel electrophoresis (150 V, 90 min). The protein bands were electro-transferred to nitrocellulose membranes (350 mA, 120 min). Membranes were blocked using tris-buffered saline (TBS) with 0.1% Tween 20 and 5% fat-free milk for 1 h. The primary antibodies were diluted in 5% BSA-tris-buffered saline containing Tween-20 (TBST) − 0.05% NaN_3_. Membranes were incubated with the primary antibody overnight at 4 °C. Subsequently, membranes were washed three times in TBST buffer, and proteins on membranes were detected using appropriate horseradish peroxidase-conjugated secondary antibodies at RT for 1 h. Results were visualized with ECL reagents. Densitometry evaluation was quantified using Image J (https://imagej.net/Open_source). The experiments were repeated with three biological replicates.

### Molecular docking

Molecular docking studies between JS-K and TAGLN^16^ were performed by AutoDock Vina version 1.1.2 (https://sourceforge.net/projects /AutoDock Vina 1.1.2-64-bit) and Autodock Tools (ADT). The X-ray crystal structure of human TAGLN was obtained from the Protein Data Bank (PDB). The structure of JS-K was obtained from the PubChem. Prior to molecular docking, the structure of TAGLN was pre-processed by adding hydrogen atoms and deleting non-essential water molecules. JS-K was converted to mol2 chemical format using Open babel. The TAGLN and JS-K were further converted to the dockable pdbqt format using Autodock tools. Docking and determination of binding affinities were carried out using AutodockVina (1.1.2) and visualized by using the PyMOL molecular graphics system (https://pymol.org/2/2).

### TAGLN silencing and cell growth assay

Our research group have constructed and maintained the TAGLN silencing cells in HepG2.2.15^17^, and they were named shTAGLN1^#^-2.15, shTAGLN2^#^-2.15 and shNC-2.15, respectively. The RNA interference target sequence (5'-GCAGGAGCATAAGAGGGAATTCACA-3' and 5'-GCAGGAGGGAAAGCATGTCAT-3') and control sequence (5'-TTCTCCG AACGTGTCACGT-3') were designed based on the RNA interference sequence design principle for TAGLN. Lentiviral shRNA vector targeting TAGLN was constructed. The TAGLN was synthesized and cloned into a lentiviral vector GV493. Monoclonal clones on the plate were selected for PCR identification, and the positive clones were sequenced and analyzed. 293 T cells were co-transfected with three plasmids (GV493, pHelper 1.0 and pHelper 2.0) to obtain lentivirus. HepG2.2.15 cells were cultured in medium containing 10 µg /mL puromycin after infected with the corresponding lentivirus. The fluorescence intensity was observed by fluorescence microscope 15 days later. The total protein was extracted and the silencing efficiency of TAGLN was verified by western blot. They were named shTAGLN1^#^-2.15, shTAGLN2^#^-2.15 and shNC-2.15^17^.

Here, cell proliferation of shTAGLN1^#^-2.15 cells and shNC-2.15 cells were evaluated with the Cell Counting Kit 8 (CCK8) assay.Briefly, these cells were seeded at 5 × 10^3^ cells/well in 96-well plates for 48 h, cultured in fresh medium containing various concentrations of JS-K. After 48 h treatment, 10 µL CCK8 was added and the plates were incubated at 37 °C for 2 h. The value of optical density (OD) of the cells was measured at 450 nm using a microplate reader (Molecular Devices, Sunnyvale,USA). The effect of JS-K on cell viability was assessed as the percentages of cell viability compared with the control cells.

### Statistical analysis

Statistics were analyzed using SPSS 17.0 software (IBM, https://www.ibm.com/products/spss-statistics). Independent sample T test and a one-way analysis of variance (ANOVA) of at least 3 biological replicates were used. *P* value < 0.05 was considered to indicate a statistically significant difference.

## Results

### Identification of significant DEPs

**Figure 1:**
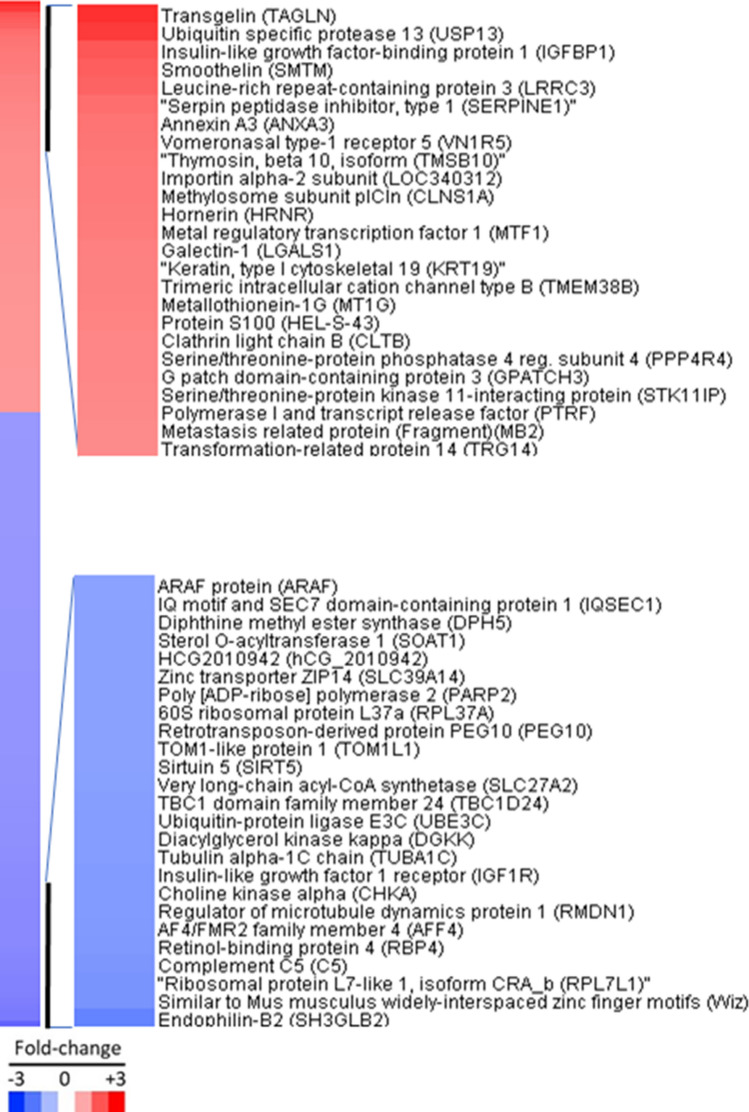
Heatmap of 182 differentially expressed proteins, with the top 25 up-regulated and 25down-regulated proteins detailed. 1.2-fold change and a P value < 0.05 (TreeView, version 1.6, https://treeview.software.informer.com/1.6/).

In order to reveal proteomic changes in HBV-positive human hepatoma cell due to JS-K, we conducted iTRAQ analysis to identify proteomic changes by comparing the DEPs. In this work, we identified 4229 protein groups from a total of 22,936 unique peptides, and, 4227 of these were quantified. Using a threshold of 1.2-fold change and a *P* value < 0.05, 182 proteins (73 upregulated and 109 downregulated) were extracted. The list of these 182 proteins was provided as Supplementary Table S2. The top 25 upregulated proteins and top 25 downregulated proteins are illustrated in the heatmap (Fig. 1). The ubiquitin specific protease 13, TAGLN and insulin-like growth factor-binding protein 1 are top 3 increased proteins, and the top 3 down-regulated proteins are Endophilin-B2, ribosomal protein L7-like 1 and complement C5.

### GO annotation of the DEPs

**Figure 2:**
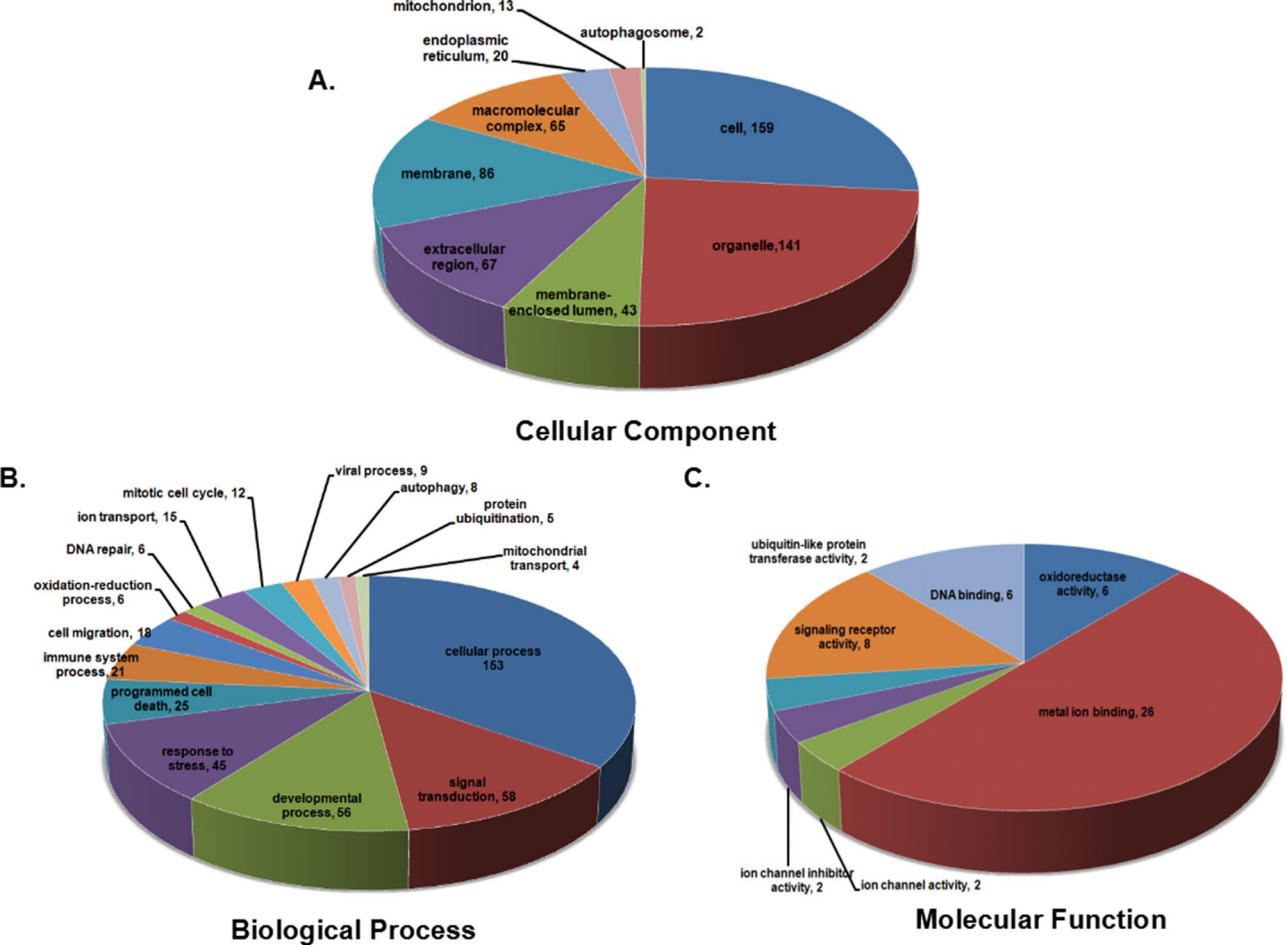
GO terms distribution. GO terms distribution in the cellular component (**A**), biological process (**B**) and molecular functions (**C**). In (**A**), the most represented categories were cell and organelle. In (**B**), cellular process and signal transduction were the most represented biological processes. In (**C**), the most represented molecular functions were metal ion binding and signaling receptor activity (http://www.geneontology.org/).

To understand the functions of the DEPs, GO analysis was carried out. All the identified DEPs were classified using the GO annotation and further categorized into three functional groups: cellular components (Fig. 2A), biological processes (Fig. 2B) and molecular functions (Fig. 2C). In the cellular components category, the results suggested most of the potential biomarkers are concentrated in the cell compartment (26.68%), organelle (23.66%) and the membrane (14.43%) compartments. In the biological process analysis, most of the DEPs participate in cellular processes (34.69%), signal transduction (13.15%), developmental process (12.70%), and response to stress (10.20%). In the category of molecular function, the DEPs were found to play a role in metal ion binding (50.00%), signaling receptor activity (15.38%), and DNA binding (11.54%), suggesting that their related functions were important in the process of JS-K against HepG2.2.15 cells.

### KEGG analysis

**Figure 3:**
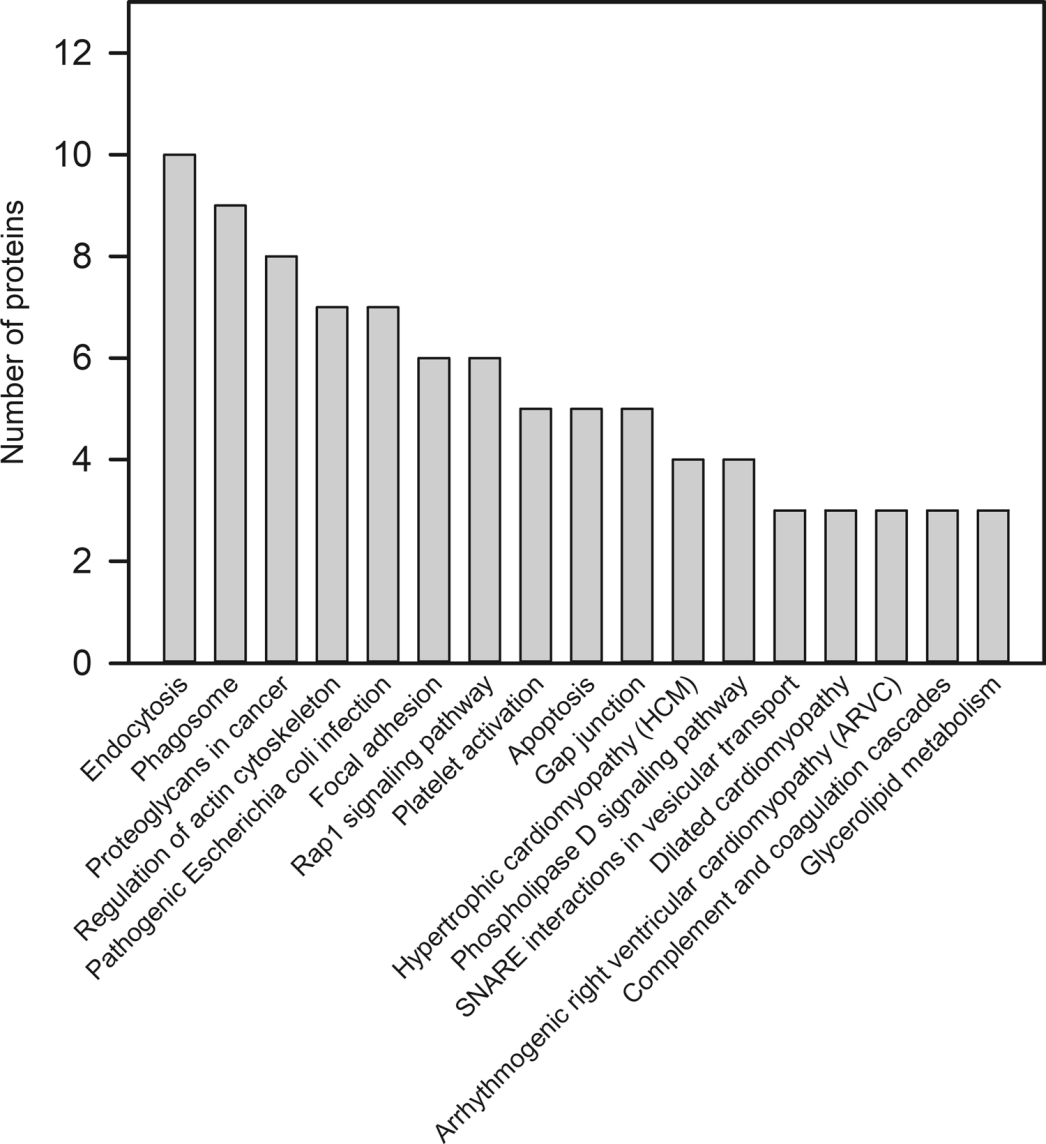
KEGG pathways analysis of differentially accumulated proteins from HepG2.2.15 cell treated with JS-K (http://www.genome.jp/kegg/pathway.html).

KEGG enrichment analysis was performed to further investigate the potential function of these DEPs. As shown in Fig. 3, these 182 DEPs were mainly mapped to 17 pathways. Ultimately, five statistically significant enrichment pathways were identified according to the hypergeometric test (*p* < 0.05). These pathways were involved in endocytosis, phagosome, proteoglycans in cancer, regulation of actin cytoskeleton and pathogenic Escherichia coli infection.

### RT-PCR and western-blot validation

**Figure 4:**
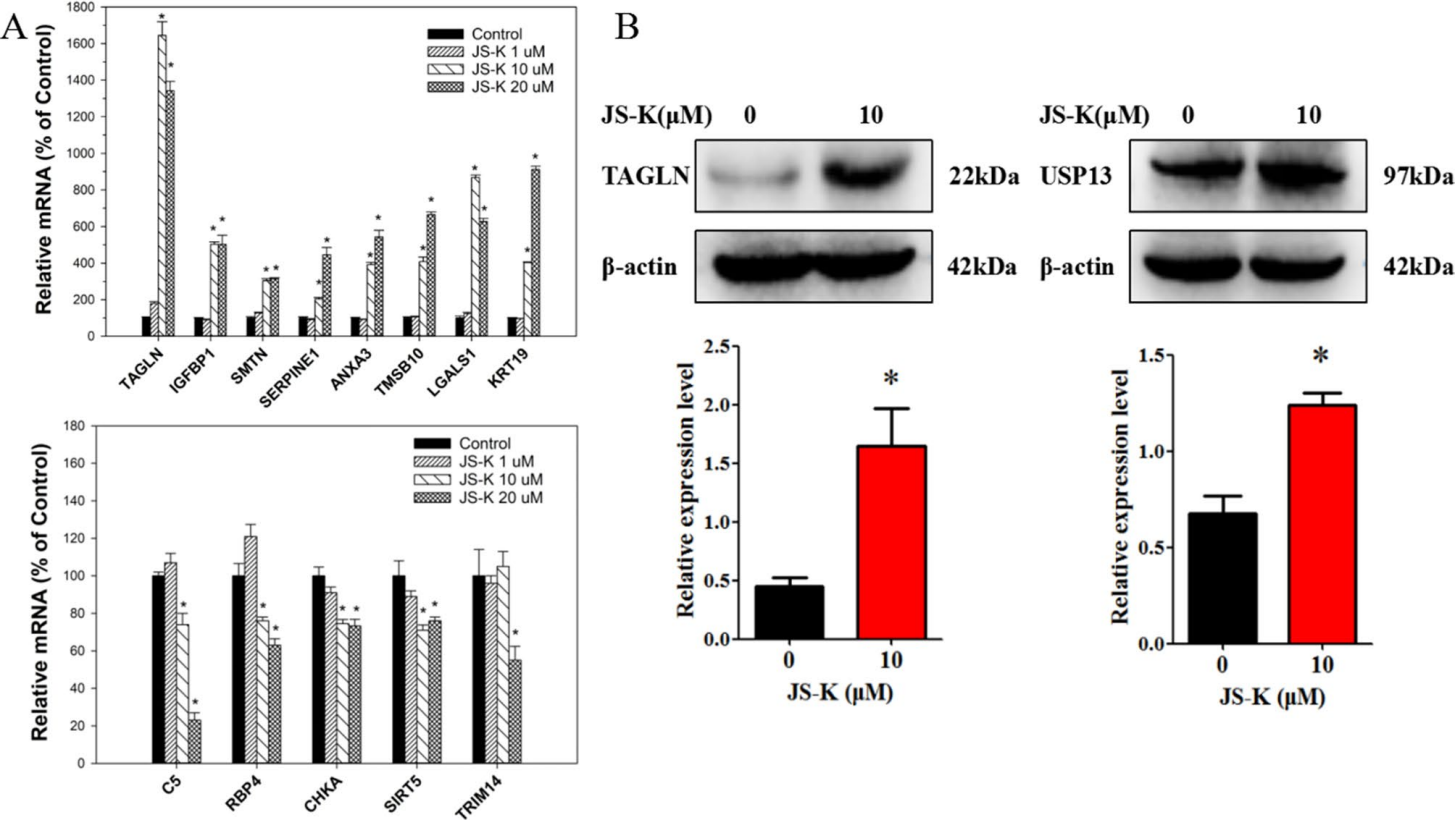
Verification of differential proteins. (**A**) The expression level of mRNA for some important genes. (**B**) Validation of the iTRAQ results using western blot. The data were expressed as the mean SD of three experiments. *P < 0.05 vs control (GraphPad, Prism5, https://www.graphpad.com).

Differential expression levels of the proteins identified by the iTRAQ approach were validated using RT-PCR analysis. JS-K treatment increased the expression of TAGLN, IGFBP1, SMTN, SERPINE1, ANXA3, TMSB10, LGALS1 and KRT19, and decreased C5, RBP4, CHKA, SIRT5 and TRIM14, consistent with proteomic results (Fig. 4A). In order to further confirm the iTRAQ results, two of the DEPs, Ubiquitin Specific Proteases 13 (USP13) and TAGLN were analyzed by western blotting. As shown in Fig. 4B, USP13 and TAGLN were significantly increased in HepG2.2.15 cells treated with 10 µM JS-K.

### The interaction between JS-K and TAGLN

**Figure 5:**
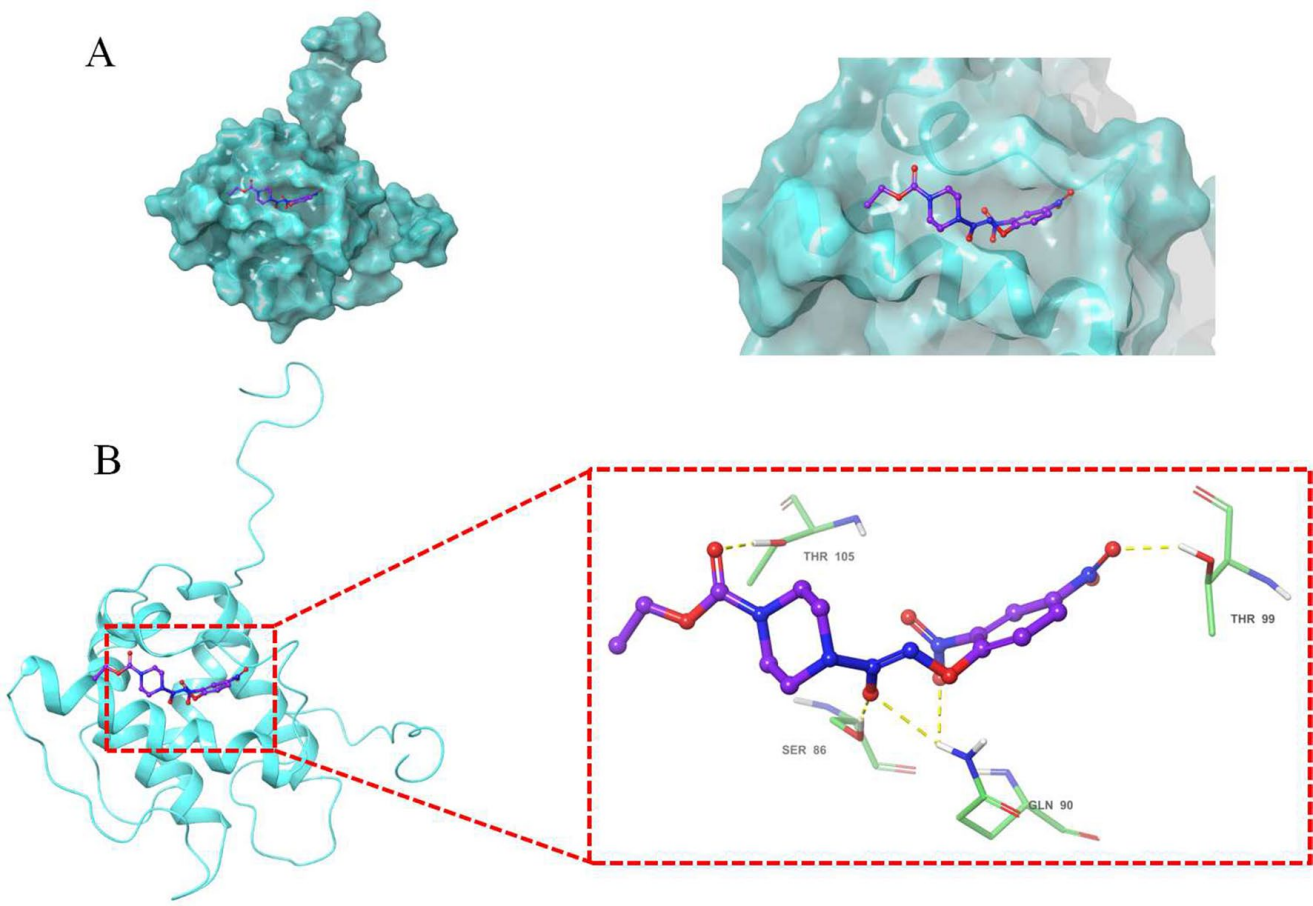
The interaction between JS-K and TAGLN were displayed using Autodock. (**A**) The substrate binding surface, (**B**) Interactions of JS-K with active site amino acids of TAGLN (AutoDock Vina, 1.1.2, https://sourceforge.net/projects /AutoDock Vina 1.1.2-64-bit).

The binding affinity of JS-K and TAGLN was determined using molecular docking method. The results showed that the strong binding affinity between JS-K and TAGLN, with binding energy of − 7.2 kcal/mol. Hydrogen bonds is an important factor that influences protein stability. As shown in Fig. 5, JS-K was entirely buried in the interior of TAGLN and JS-K forms a hydrogen bond network with the four amino acids Thr 105, Ser 86, Gln 90 and Thr 99 near the active site of the protein, respectively.

### TAGLN silencing reversed the anti-tumor effect of JS-K in HepG2.2.15 cells

**Figure 6:**
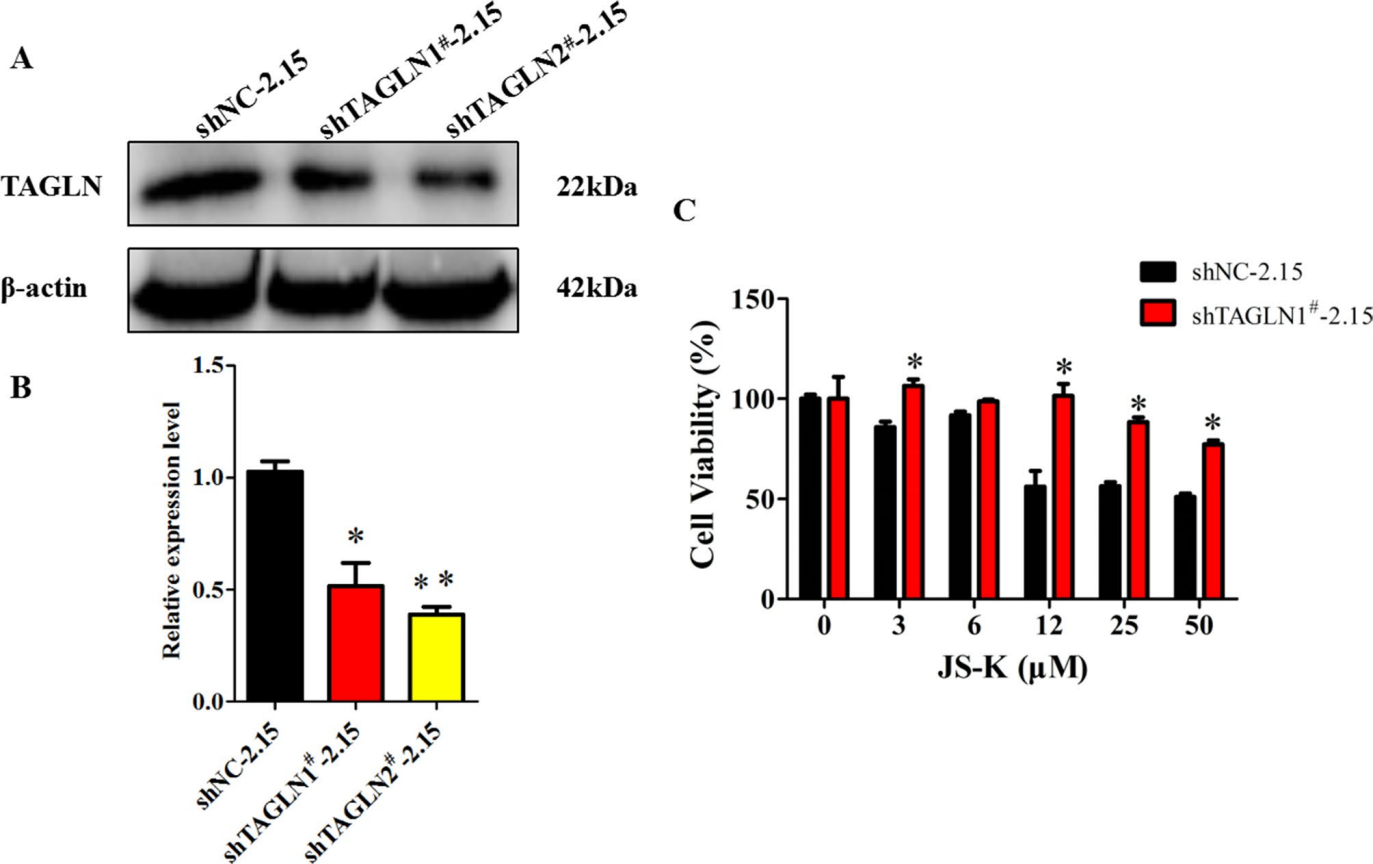
The Effect of TAGLN silencing on the viability of HBV-positive liver cancer cells treated with JS-K. (**A**,**B**) Expression of TAGLN in shTAGLN1^**#**^-2.15 and shTAGLN2^**#**^-2.15 cells by western blot. (**C**) Cells were cultured in the different concentration of JS-K for 48 h, cells viability were determined by the CCK8 assay. The data were expressed as the mean SD of three experiments *P < 0.05, **P < 0.01 vs shNC-2.15 (GraphPad, Prism5, https://www.graphpad.com).

Our published results confirm that TAGLN expression increased in HBV-positive hepatocellular carcinoma tissues and cells compared with HBV-negative hepatocellular carcinoma^17^. In order to evaluate the effects of TAGLN on cell viability of HepG2.2.15 cells treated with JS-K, shNC-2.15 and shTAGLN1^#^-2.15 cells were used (Fig. 6A,B)^17^. As shown in Fig. 6C, the cell viability gradually decreased with the increase of JS-K concentration by the CCK8 assay at 48 h. At the same concentration, the cell viability of shTAGLN1^#^-2.15 group was higher than the control group (*P* < 0.05). It suggested that the anti-tumor effect of JS-K is significantly weakened when TAGLN is knocked down in the HepG2.2.15 cells.

## Discussion

JS-K has been received a lot of attention because of its highly effective and broad-spectrum anti-tumor effects^2^. Our previous work demonstrated that JS-K induces DNA damage and apoptosis in hepatoma cell lines Hep3B cells and HepG2.2.15 cells^6,9^ and decreased the expression of HBsAg and HBeAg in HepG2.2.15 cells^8^. The present study successfully utilized the iTRAQ technology to identify 182 differentially expressed proteins. They are mainly localized in the organelle, membrane, extracellular region. The biological processes involved mainly include cellular processes, signal transduction and developmental process. The enrichment analysis of KEGG revealed that these differential proteins were mainly enriched in 5 KEGG pathways, including endocytosis, phagosome, regulation of actin cytoskeleton, rap1 signaling pathway and apoptosis. Fourteen of the top differentially expressed proteins were confirmed by qPCR and/or western blot analysis. This is the first attempts to explore molecular mechanisms of JSK against HBV-positive HepG2.2.15 cells.

TAGLN is a shape change-sensitive 22 kDa actin-binding protein of the calponin family. It is ubiquitous to vascular and visceral smooth muscle and is an early marker of smooth muscle differentiation^16,18^. TAGLN is implicated in cancer development and upregulated in stromal cells of lymph node positive breast cancer^19,20^, but it is also reported to be a tumor suppressor, especially in liver cancers^16–18,21–23^. As a tumor suppressor, TAGLN has been shown to inhibit prostate cancer cell growth^18^. TAGLN could attenuate the proliferation and invasive ability of human colon adenocarcinoma cell line LoVo cells and enhances LoVo cell apoptosis^22^. We found JS-K could enhance the expression of TAGLN mRNA and protein levels, indicating JS-K could inhibit HCC progression. Next, we test the binding affinity of JS-K and TAGLN by using molecular docking method. The results showed that the strong binding affinity between JS-K and TAGLN. Hydrogen bond is an important factor that influences protein stability. Our results showed that the JS-K was entirely buried in the interior of TAGLN. JS-K forms a hydrogen bond network with the four amino acids Thr 105, Ser 86, Gln 90 and Thr 99 near the active site of the protein, respectively. These hydrogen bond interactions promote the stable binding of JS-K to the active site of TAGLN. Our published results confirmed that TAGLN expression increased in HBV-positive hepatocellular carcinoma tissues and cells compared with HBV-negative hepatocellular carcinoma, and interference with TAGLN expression can inhibit the proliferation of HepG2.2.15 cells and weaken their ability of migration and invasion^17^. The result of CCK8 assay suggested that the anti-tumor effect of JS-K is significantly weakened when TAGLN is knocked down in the HepG2.2.15 cells. In combination with the results of molecular docking, it is a strongly hint that the anti-tumor effect of JS-K is achieved by binding to TAGLN. The upregulation of TAGLN by JS-K could be critical in JS-K antitumor effects.

USP13 belongs to the deubiquitinating enzyme (DUB) superfamily and is implicated in tumorigenesis by deubiquitinating tumor suppressors p53, phosphatase and tensin homologue deleted on chromosome 10 (PTEN) and MITF^24^. As a STING-interacting protein that catalyzes de-ubiquitination of STING, USP13 deconjugates polyubiquitin chains from STING and prevents the recruitment of TBK1 to the signaling complex, thereby regulating cellular antiviral responses^25^. USP13 stabilizes tumor suppressor PTEN via direct binding and deubiquitinating of PTEN, and functions as a tumor suppressing protein^26^. USP13 also positively regulates interferon (IFN) signaling by deubiquitinating and stabilizing STAT1 protein and plays a role in the antiviral activity of IFN against DEN-2 virus replication^27^. Thus, USP13 induction by JS-K could inhibit the development and progression of HBV-positive HCC.

HBV infection is associated with HCC development. In HBV replicating HepG2 cells, the expression of insulin-like growth factor-binding protein 1 (IGFBP1) was markedly suppressed^28^. In patients infected with hepatitis C virus (HCV), the expression of smoothelin (SMTN) was also markedly decreased^29^. In the current study, JS-K significantly increased these two proteins as evidenced by iTRAQ analysis and confirmed by qPCR, suggesting these two proteins could be important in JS-K anti-tumor and antivirus effects in HepG2.2.15 cells.

Platycodin D, a triterpenoid saponin isolated from Platycodonis Radix, is a famous Chinese herbal medicine that has been shown to have anti-cancer effects in several cancer cells. The expressions of Galectin-1 (LGALS1) and Keratin, type I cytoskeletal 9 (KRT9) protein were up-regulated in Platycodin D-treated in HepG2 cells^30^. In the present study, JS-K treatment significantly increased LGALS1 and KRT19. In the HBsAg-HBV transgenic mouse model, KRT19 was increased in the early stage of HCC, but in the late stage of HCC, it was decreased^31^. JS-K induction of these proteins could play a role in its anti-cancer effects in HepG2.2.15 cells.

The leaf extract of Tamarindus indica L. had been reported to possess antioxidant activities, and increase SERPINE1 leading to decreased expression of plasminogen activator inhibitor type-1 (PAI-1) protein, a major physiologic inhibitor of fibrinolysis^32,33^. Induction of SERPINE1 by JS-K could inhibit liver fibrosis and the liver fibrosis progression to HCC. Annexin A3 (ANXA3) is a biomarker of HCC^34^, its overexpression is associated with multi-drug resistance (MDR)^35^. JS-K is reported to overcome MDR to enhance metallotherapy^36^ and reversal of drug resistance in human MDR tumor cells^37^ Thymosin β10 (TMSB10) has been demonstrated to be overexpressed and function as an oncogene in most types of human cancer including HCC^38^. In the present study, the expression of both proteins was increased by JS-K. One of the tentative explanations could be that JS-K further induced cellular stress, and these two proteins act as acute phase proteins for tumor cells to adapt the challenge. This hypothesis needs further investigation.

Among differentially expressed proteins, 109 were decreased, and these proteins are also associated with HCC progression. For example, the Complement C5 (C5) controls liver lipid profile, promotes liver inflammation and HCC development^39^. The anticancer agent Emodin inhibited the growth of HCC cells in vitro and in vivo, and markedly decreased the expression of 712 genes in HepG2 cells including C5 mRNA^40^. Sirtuin-5 (SIRT5) belongs to a family of NAD^+^-dependent lysine deacetylases called Sirtuins. SIRT5 knockdown suppresses HCC cell proliferation and SIRT5 overexpression promotes HCC cell proliferation, and downregulation of SIRT5 has been proposed as a drug target for HCC therapies^41^.

Retinol-binding protein 4 (RBP4) is the major transport protein for retinol and also acts as hepatotropic factor associated with HCC progression. Endosialin limits HCC progression via suppression of RBP4, IGF2, DKK1 and CCL5^42^. Choline kinase (CHKA) is the first enzyme in the CDP-choline pathway for the synthesis of phosphatidylcholine, Inhibition of CHKA activity has been proposed as a target for cancer therapies and miR-876-5p transfection decreased CHKA in HepG2 cells^43^. Tripartite motif containing 14 (TRIM14) has been reported to play a critical role in tumor development and high levels of TRIM14 are associated with poor prognosis in HCCpatients,and downregulation of TRIM14 could be beneficial in JS-K antitumor effects^44^. We have used qPCR to confirm the expression of these 5 proteins. Fortunately, JS-K treatment significantly decreased the expression of these HCC progression-associated proteins, adding mechanistic evidence for JS-K against HepG2.2.15 cells.

From analysis of the above differentially expressed proteins, it is clear that JS-K has both anti-tumor and anti-virus effects, making it unique against HBV-positive HepG2.2.15 cancer cells. It should be noted that the antitumor effects and anti-HBV effects are not mutually exclusive, and JS-K effects are not limited to these 14 proteins either. All JS-K induced changes could act in an integrative manner to exert antitumor effects against HBV-positive HCC.

In summary, our study obtained many DEPs in JS-K anti-HBV-positive HCC cells through iTRAQ technology combined with mass spectrometry. TAGLN could be an important target in JS-K anti-HBV-positive liver cancer cells. These proteomic findings will provide some new targets for the treatment of HBV-positive HCC.

## Supplementary Information

Supplementary Information 1.

Supplementary Information 2.

Supplementary Information 3.

Supplementary Information 4.

## Data Availability

The datasets generated during this study were deposited to the ProteomeXchange Consortium (http://proteomecentral.proteomexchange.org) via the iProX partner repository 61 with the dataset identifier IPX0002754001. The list of differentially expressed proteins was included in Supplementary Table S2.
