# Supplementary material for: Identification of targets of JS-K against HBV-positive human hepatocellular carcinoma HepG2.2.15 cells with iTRAQ proteomics
Source: Sci Rep. 2021 May 17;11:10381. doi: 10.1038/s41598-021-90001-3 (PMC8129129; doi:10.1038/s41598-021-90001-3)
Supplement: Supplementary file 4 — Supplementary Information 4. [file 41598_2021_90001_MOESM4_ESM.pdf]

Identification of targets of JS-K against HBV-positive human hepatocellular carcinoma HepG2.2.15 cells with iTRAQ proteomics

Zhengyun Liu, Yan Xu, Wanling Zhang, Xinghong Gao, Guo Luo, Hong Song, Jie Liu, Huan Wang

Fig.4 β-actin

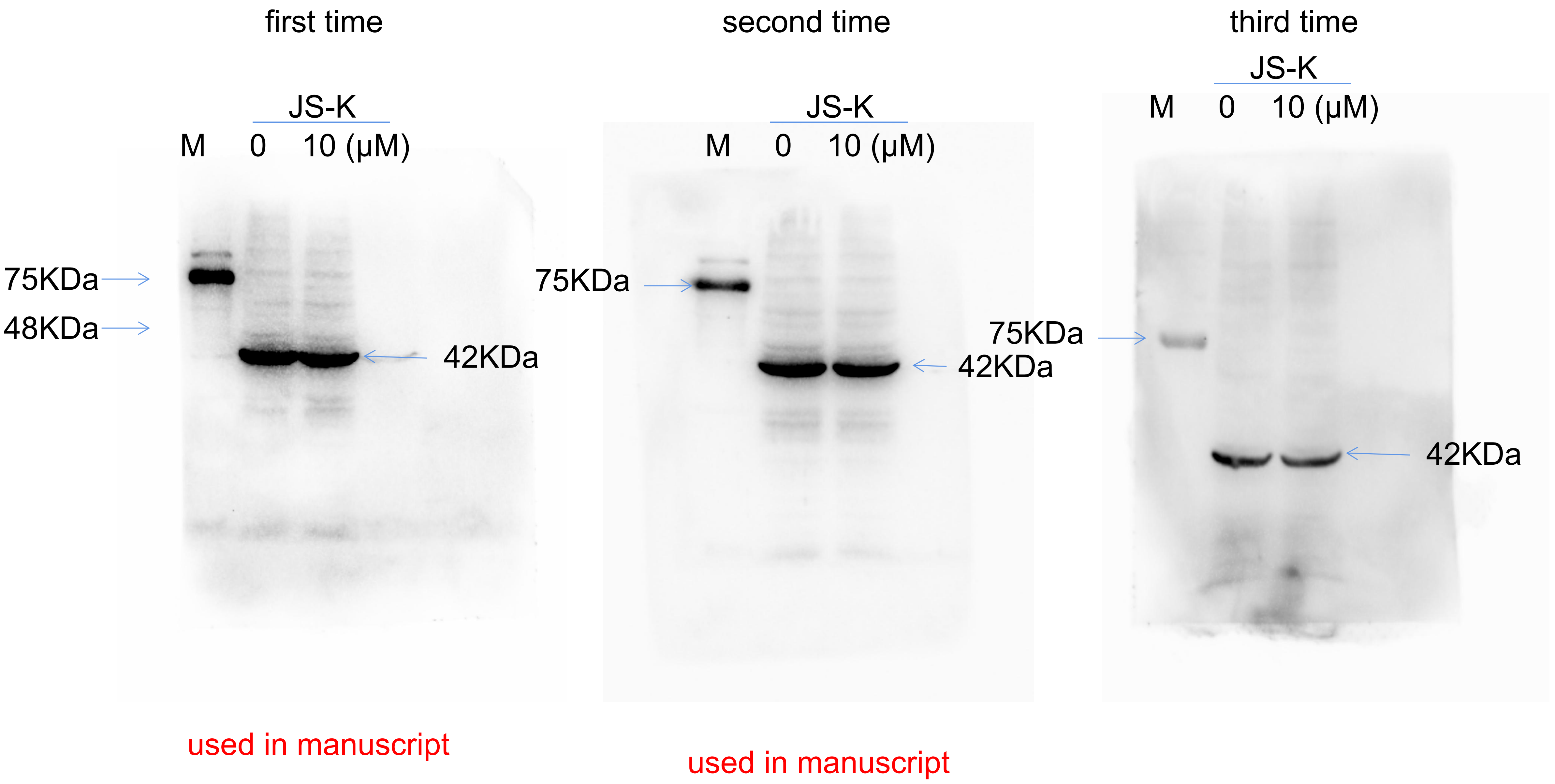
first time
JS-K
M 0 10 (μM)
75KDa
48KDa
42KDa
second time
JS-K
M 0 10 (μM)
75KDa
42KDa
third time
JS-K
M 0 10 (μM)
75KDa
42KDa
used in manuscript
used in manuscript

Fig.4 USP13

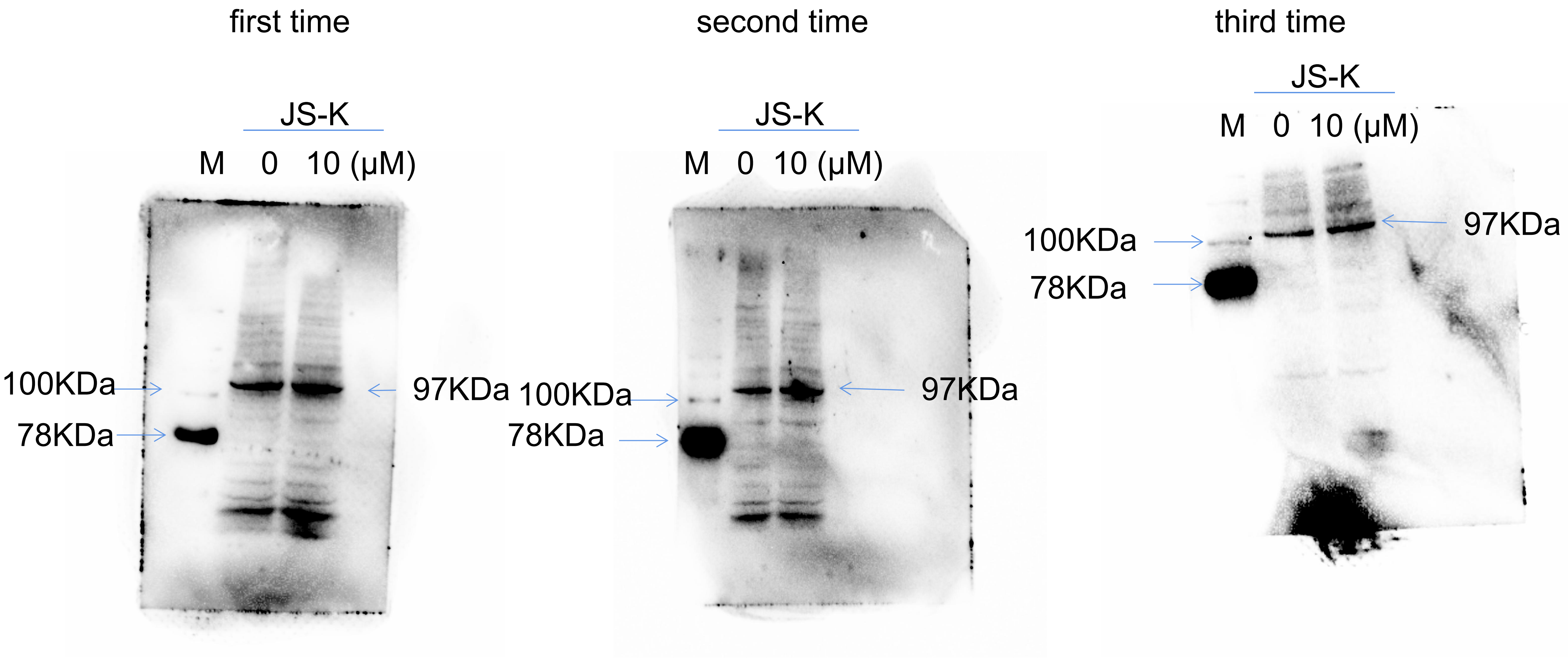

used in manuscript

Fig.4 TAGLN

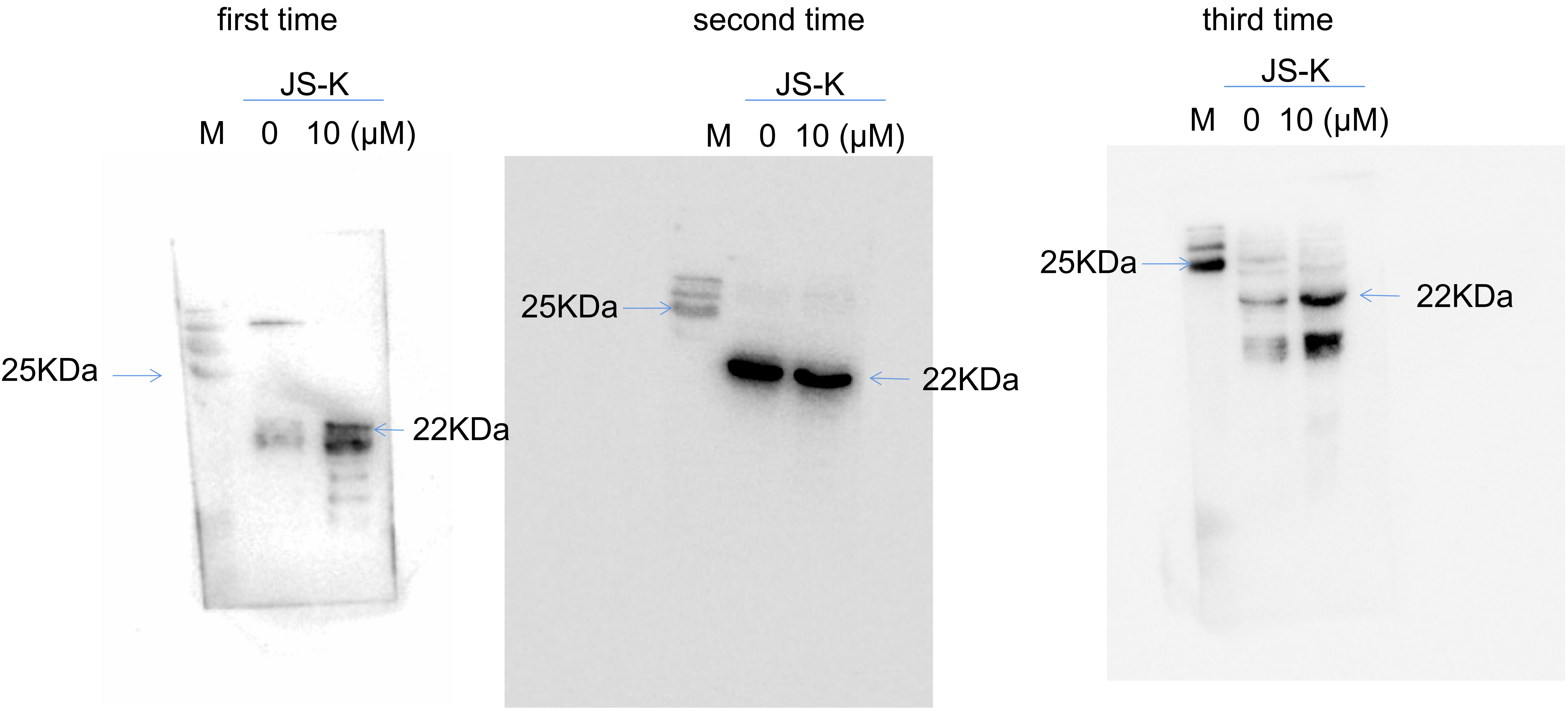

used in manuscript

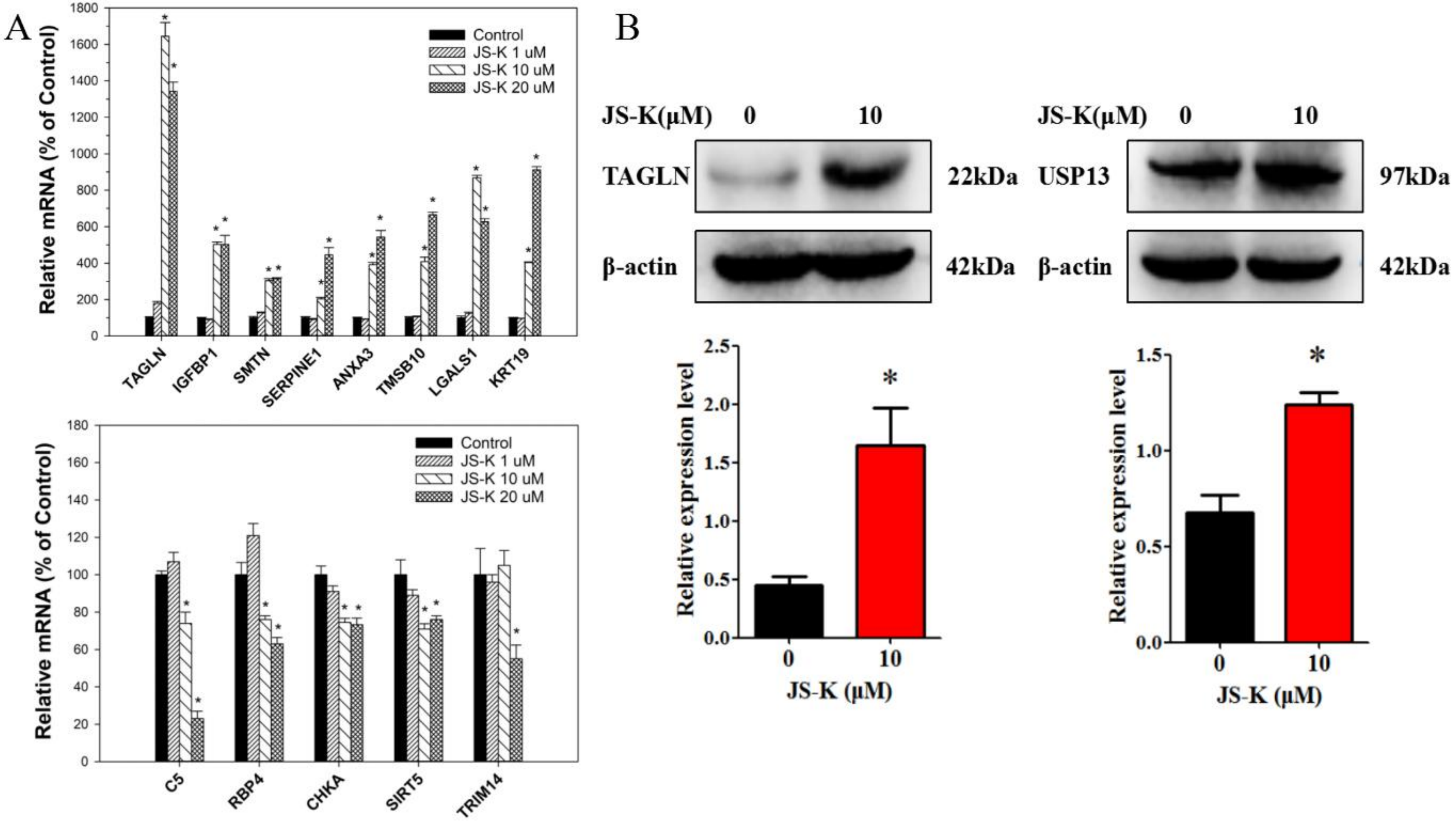

Figure 4. Verification of differential proteins.

Fig.6

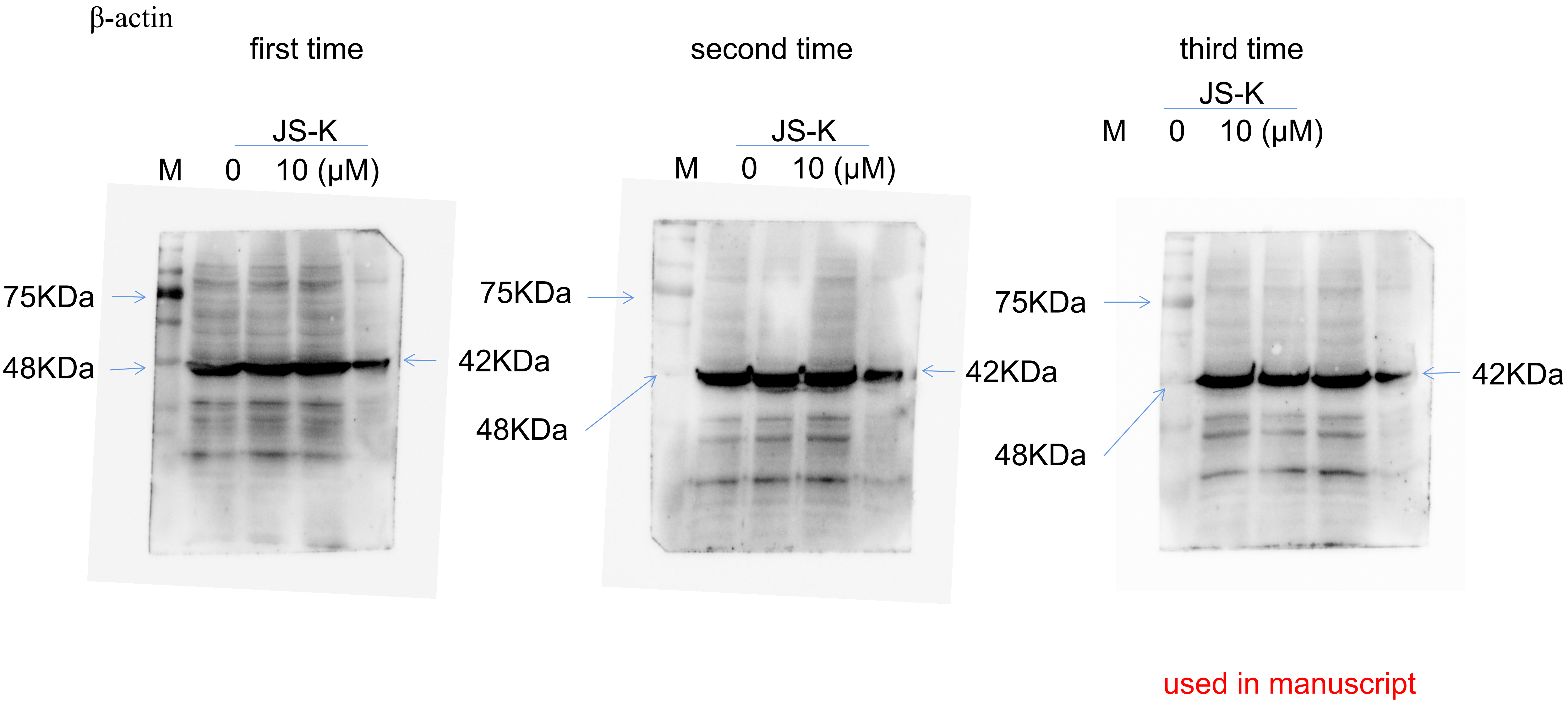
β-actin
first time
JS-K
M 0 10 (μM)
75KDa
48KDa
42KDa
second time
JS-K
M 0 10 (μM)
75KDa
48KDa
42KDa
third time
JS-K
M 0 10 (μM)
75KDa
48KDa
42KDa
used in manuscript

Fig.6

TAGLN

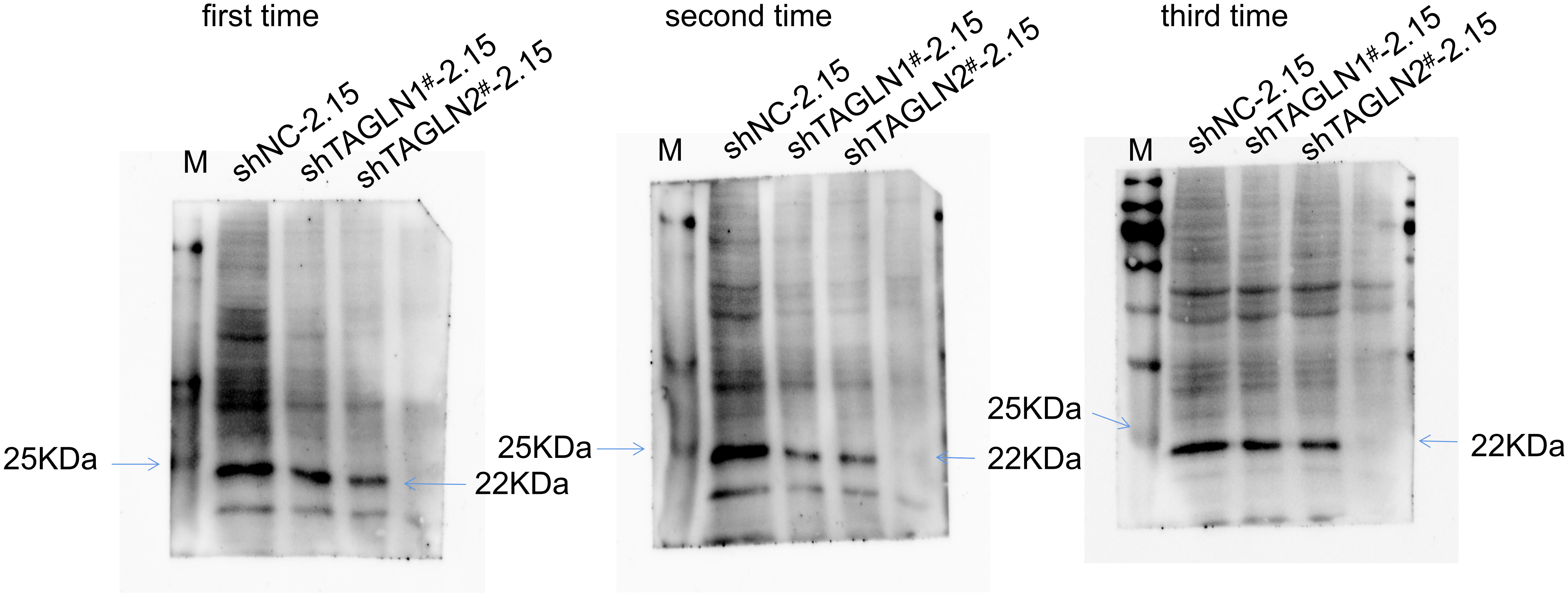

used in manuscript

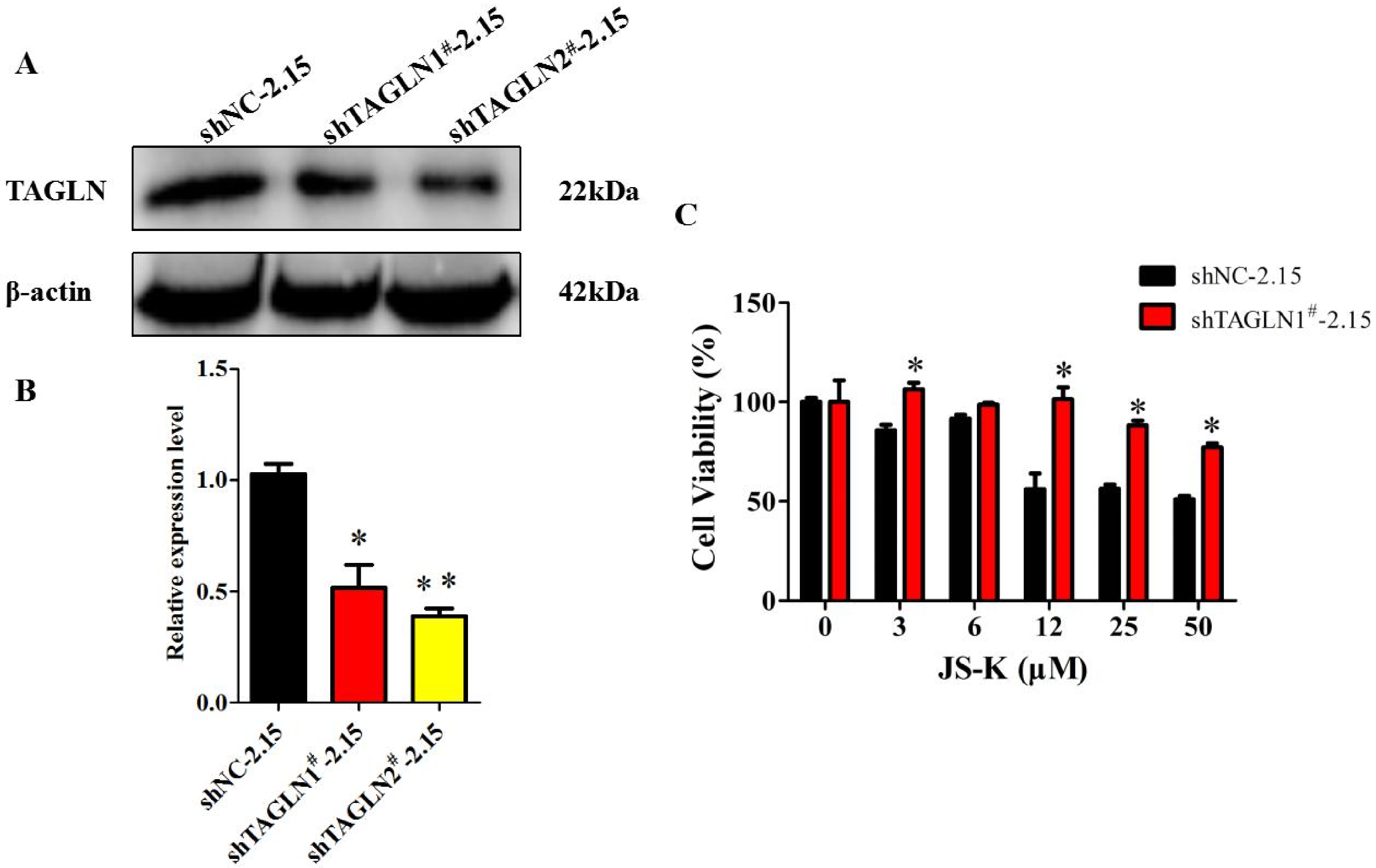

Figure 6. The Effect of TAGLN silencing on the viability of HBV-positive liver cancer cells treated with JS-K.
